# Exploring Teacher and Parent Perspectives on School-Based Masculinities in Relation to Mental Health Promotion

**DOI:** 10.3389/fpsyg.2022.864124

**Published:** 2022-06-13

**Authors:** Michael J. Wilson, Kate Gwyther, Magenta Simmons, Ray Swann, John L. Oliffe, Kate Casey, Simon M. Rice

**Affiliations:** ^1^Orygen, Parkville, VIC, Australia; ^2^Centre for Youth Mental Health, The University of Melbourne, Parkville, VIC, Australia; ^3^Crowther Centre, Brighton Grammar School, Brighton, VIC, Australia; ^4^Department of Surgery, Royal Melbourne Hospital, The University of Melbourne, Parkville, VIC, Australia; ^5^School of Nursing, University of British Columbia, Vancouver, BC, Canada; ^6^Department of Nursing, The University of Melbourne, Parkville, VIC, Australia

**Keywords:** masculinity, adolescent, mental health, intervention, school

## Abstract

The capacity for boys’ and young men’s mental health promotion to act *via* shifting masculine norms that are linked to poor mental health outcomes, highlights the need to improve the extent to which school-based programs can promote mental health through leveraging more positive embodiments of masculinity. To-date, the perspectives of parents and teachers on such processes are understudied. This qualitative study presents teacher and parent views regarding adolescent masculinities and avenues for school-based developmental programming for boys and young men. In this study, 16 individual qualitative interviews were undertaken with 10 parents (six females, four males), and six teachers (three females, three males), recruited from an independent all-boys’ grammar school in Melbourne, Australia. Thematic analysis of parents’ and teachers’ perspectives indicated their perception of the role of context-dependent “public” and “private” masculinities, the influence of Australian masculinity norms, and the role of private boys’ school cultures in the development of adolescent masculinities. Additionally, strategies for development encompassed participants’ appetite for boys’ exposure to positive role models, in addition to consistent and relevant developmental programming to support positive masculinity development. Findings have implications for efforts to support prosocial masculine identity development *via* school-based initiatives, as an avenue to promoting mental health of boys and young men.

## Introduction

Suicide rates among boys and young men are approximately triple those observed in young women (World Health Organisation, 2014). Australian data indicate age-specific suicide rates (per 100,000 population) among 15–19-year-olds to be 16.9 for young men and 6.1 for young women; and among 20–24-year-olds, 24.9 for young men and 7.3 for young women (Australian Bureau of Statistics, 2020). This cements the need to explore the varied influences on the proliferation of suicidality in boys and young men. A wealth of research has connected behavioral norms grounded in so-termed “traditional masculinity” with low rates of help-seeking for emotional problems in boys and young men compared to girls and young women (Slade et al., 2009), alongside evidence associating adherence to masculine codes of self-reliance with risk of suicidal thinking (King et al., 2020). Importantly, boys’ and young men’s perception that they are *expected* to behave in traditionally masculine ways (i.e., avoiding help-seeking in favor of self-reliance) by the people around them can be a driving force in sustaining these norms (Irvine et al., 2018). As such, further research is needed to uncover the social processes and contexts that continue to underpin boys’ and young men’s perceptions of the expectation to behave in accordance with traditional masculinity. Findings in this domain will help to refine school-based mental health promotion programs that aim to norm healthy prosocial embodiments of masculinity as a conduit to promoting boys’ and young men’s mental health.

### Adolescent Masculinities

Social constructionist perspectives suggest that masculinities (i.e., the various ways of being and expressing one’s gender as a boy or man) are learnt behaviors, attitudes and norms that define what it is to “be a man” (Connell, 1995). These meanings vary across cultures and contexts, and in-turn influence mental health outcomes in myriad ways (Robertson, 2007; Gough, 2018). In this sense, masculinity is not a homogenous concept, but rather, as proposed by Connell and Messerschmidt (2005), masculinities exist in multiple forms explicating the diversity of gendered expressions between and within cultures, time and individuals. Membership of varying hierarchical social categories also intersects with the embodiment of masculinities, such that, for example, variation across class, race, and sexualities (i.e., identification as heterosexual or a sexual minority such as gay or bisexual) will influence practices and performative behaviors in the enactment of masculinities, and in turn, mental health (Griffith, 2012).

There is long-standing recognition that rigid conformity to masculine norms can contribute to psychosocial and health-related problems among boys and young men (Wong et al., 2017; Rice et al., 2018). Abundant literature has linked rigid adherence to “traditional” masculine norms (e.g., stoicism, toughness, emotion suppression, and power over women) to a range of negative outcomes, including aggression, violence, substance misuse, reduced wellbeing, low help-seeking, and school disengagement (Young et al., 2002; Levant et al., 2009; Ueno and McWilliams, 2010; Wong et al., 2017; Ravn, 2018). Moreover, research has highlighted associations between conformity to norms of violence and self-reliance with risk of suicidal thinking in boys and young men (King et al., 2020). Boys who experience greater pressure to enact their masculine gender role are also more likely to experience depression, and in turn, increasingly likely to report suicide ideation (O’Beaglaoich et al., 2020).

During adolescence, boys and young men may experience heightened pressure to display or embody masculine ideals perceived as socially condoned in order to mark their difference from the embodiment of femininities (Connell, 1995). Put another way, boys and young men are often socialized to defend or contest perceived or actual judgment of their behaviors and identity aligning with feminine norms by proving their alignments to traditional masculinity (Vandello and Bosson, 2013). The foundation of masculinity is thought to be the rejection of that which is not masculine; the divergence of oneself from femininities or subordinate masculine identities (e.g., sexual minority men; Connell, 1995). This thinking has culminated in emerging understandings of the extent to which a certain archetype of masculinity can be policed for boys, such that they can suffer a sense of reduced social standing if they are perceived to violate the “norm” when it comes to enacting one’s masculinity (Reigeluth and Addis, 2021). In the context of suicidality, the construction of help-seeking and relying on others as indicative of weakness or femininity in boys and young men is thought to, at least in part, precipitate reduced propensity to disclose distress in order to uphold an image of masculine self-reliance (Möller-Leimkühler, 2002; Seidler et al., 2016). While some studies indicate that adherence to masculinity norms may peak during adolescence around ages 15–18 (Rice et al., 2011; Herreen et al., 2021), researchers have also noted increasing flexibility in the enactment of masculinity norms by younger generations of boys and young men (Anderson and McCormack, 2018). Contemporary male youth in the global north are commonly thought to be engaging in a process of identity formation which requires a complex negotiation of modern-day expectations (e.g., engagement with feminism; help-seeking for emotional problems) against the backdrop of traditional masculinity expectations (e.g., power over women; emotional restriction and rigid self-reliance; Elliott, 2019). Crucially, therefore, understanding the contextual influences on this process of masculine identity expression enables a critique of the ways in which this traditional archetype of masculinity continues to influence the mental health of boys and young men.

### School-Based Masculinity Development

One setting identified as critical to this process of masculine identity construction is the secondary school environment. Schools are key sites for the production and negotiation of male-coded behaviors, where certain features (e.g., the separation of male and female students in single sex schools) can influence enactments of masculinity (Martino and Meyenn, 2001). Students navigate a variety of formal structures (e.g., curriculum, policy) and informal structures (e.g., relationships with students, teachers) through which behavioral norms are regulated and enforced (Swain, 2006). Influences on adolescent masculinity development can permeate various interdependent settings, with secondary school as the epicenter. For instance, behavioral norms and expectations set in the home through parental modeling (and indeed those set in the home of one’s peers) may permeate the school; and *vice versa*, where teachers can also model masculinities (Hickey and Mooney, 2018). Equally, parenting efforts to shape prosocial masculinities can be thwarted within the school environment, where peer-driven policing of adherence to traditional masculinities is strongest (Reigeluth and Addis, 2016). Parents, teachers and boys’ and young men’s peers are therefore all active agents in the development of boys’ gender concepts and understanding of their own masculinities (Connell, 1996; Odenweller et al., 2013; Bishop, 2017). Given this, understanding the perspectives of all parties is vital to appraising how masculinity is learnt, performed, enacted, and reinforced by boys and those around them in school settings (Weaver-Hightower, 2003; Hickey and Mooney, 2018). A large body of research has explored boys’ and young men’s experiences of the “policing” of adherence to traditional masculine norms in the school context (Reigeluth and Addis, 2016) where embodiment of “masculine” attributes is deemed a social “must” (MacLean et al., 2010). Yet to complement this literature, further research aiming to canvass the perspectives of parents and teachers on adolescent masculine identity development, and how these parties can best support gains made *via* school-based mental health promotion interventions, is warranted. Moreover, single-sex schools simultaneously occupy a position wherein traditional masculine norms can be both challenged and inadvertently reinforced; equity-driven, staff-, and student-led initiatives to encourage a wider school climate grounded in positive, prosocial masculinities could be a key agent of change here (Hickey and Mooney, 2018).

Building on a long-standing call for psychoeducational programs for boys and young men that aim to target restrictive masculine socialization (O’Neil and Luján, 2009), interventional research is beginning to capitalize on the school environment as a prime avenue for programs intended to shift the pervasiveness of traditional masculinity norms for boys and young men. One example in Australia is the Silence is Deadly program, recently evaluated by Calear et al. (2021). Delivered to boys and young men in Australian secondary schools, the program aims to provide psychoeducation regarding men’s mental health, suicide and help-seeking, while communicating explicitly the role that adherence to traditional masculinity norms such as self-reliance and emotional restrictiveness can play in shaping negative attitudes toward help-seeking (Calear et al., 2017). Whilst positive effects of the program on help-seeking intentions were observed (Calear et al., 2021), there is scope to improve the extent to which such programs can achieve the complicated goal of shifting identification with masculinity norms. Researchers, boys and young men alike have long called for the need for positive, strength-based programming that does not simply tell boys and young men what not to be, but rather achieves this end indirectly *via* encouragement of a new, positive and prosocial archetype of masculinity that, for example, embraces enlisting professional help as indicative of psychological strength (Kiselica et al., 2016; Wilson et al., 2021a). Given shifting identification with masculinity norms is a long-term process involving multiple agents of influence across the school context (i.e., peers, parents, and teachers; Martino and Meyenn, 2001; Marmion and Lundberg-Love, 2004; Martino, 2008) further research is needed to explore the level of understanding of parents and teachers in school-based masculinity development. This will help to inform efforts to subsequently involve parents and teachers in mental health promotion interventions that are grounded in promoting healthy embodiments of masculinity.

### The Present Study

While researchers have commonly discussed male students influencing role norm adherence of their peers in a student body (Reigeluth and Addis, 2016; Rogers et al., 2021), the instrumental roles of teachers and parents in male students’ masculine identity development are under-researched. Foundational research has noted the influence of teachers on masculinity development among male students, particularly in the context of physical education where it has been suggested that male teachers in particular reproduce the norms characteristic of traditional masculinity, given its association with sporting prowess (Jackson, 2010; White and Hobson, 2017). Findings regarding the respective roles of parents and teachers in school-based masculine socialization will be readily translatable to school-based health promotion interventions designed to encourage positive masculinity (i.e., developmental progress toward the embodiment of human strengths by males; Wilson et al., 2021a) as a means to benefit mental health. Finally, regarding a worthy context to investigate parent and teacher perspectives on school-based masculinity development, private all-boys schools have been long discussed in the literature as presenting a unique context for the competitive enactment of hyper-masculinity among boys and young men (Hickey, 2010); yet the perspectives of the parents’ and teachers connected to these schools remain under-researched to date.

As such, the aim of the current study was to investigate parents’ and teachers’ perceptions of masculinity development in an all-male private school context, and their views regarding priorities for school-based initiatives designed to support boys toward positive trajectories of masculine identity development. The secondary aim was to uncover potential links with school-based mental health promotion grounded in shifting masculine norms. One overarching research question was addressed: What are teacher and parent perspectives of masculinities in secondary school boys attending an all-male school?

## Materials and Methods

### Design

The study applied a qualitative design using individual interviews to explore the attitudes of parents and teachers toward masculine identity development among boys and young men. Study reporting adheres to the consolidated criteria for reporting qualitative research reporting guidelines (Tong et al., 2007).

### Participants and Context

The sample comprised 16 individuals, including 10 parents (six female, four male; all with a son in year 11) and six teachers (three female, three male), from a high-fee independent all-boys’ grammar school in Melbourne, Australia.

To contextualize the setting in light of existing scholarship regarding gendered school curriculum, during June 2014-January 2015 the leadership team at the school explored academic evidence regarding biological determinants of learning differences that could be incorporated into the teaching and learning programs. Among the sources was a synthesis of meta-analyses by Hattie (2008) wherein gender differences in learning styles (comparing boys and girls) concluded “the differences between males and females should not be of major concern to educators. There is more variance within groups of boys and within groups of girls than there are differences between boys and girls” (p. 56). The school’s approach to wellbeing-science programs aims to personalize school-based wellbeing by applying a gendered lens to the PROPSER framework for incorporating positive psychology principles in education settings (Noble and McGrath, 2015). This approach aims to do justice to the culturally determined factors (i.e., boys’ gender socialization) that impacts mental health promotion for boys and men (Rice et al., 2021), especially in education settings (Salmela-Aro, 2014).

Participants were recruited *via* convenience sampling, but where possible recruitment aimed to include participants diverse in role (i.e., parent or teacher) and age range. This study involved individuals who were recruited as part of a larger study evaluating a school-based rite of passage program for boys and young men (Wilson et al., 2021b), and had indicated their consent to be contacted for future research. The program was a father (or father figure) and son experiential program where participants were encouraged to reflect on their experiences of gendered norms, and, for participating boys, the kinds of men they wished to develop into. All participating fathers and male teachers in this study had participated in the program, and recruitment was also extended to mothers and female teachers in attempt to achieve an even gender distribution of participants. Email invitations that detailed the study description and aims were sent by associated study personnel employed at the school, to parents and teachers. Emails were sent to 38 potential participants in total, resulting in a response and consent rate of 42 per cent. Participants were aged between 32 and 73 years (*M* = 49.19, *SD* = 10.35). All participants identified their sexual orientation as heterosexual. Nine participants were born in Australia, and seven were born overseas (United Kingdom = 5, New Zealand = 2). Demographic characteristics of all participants are detailed in Table 1 below.

**TABLE 1:** Summary of participant demographics.

Demographic	Statistic
Age (range, *M*, *SD*)	32–73 years, 49.19, 10.35
**Gender (%, *n*)**	
Man	43.8 (7)
Woman	56.2 (9)
**Sexual orientation (%, *n*)**	
Heterosexual	100.0 (16)
**Born overseas (%, *n*)**	
Yes	43.8 (7)
No	56.2 (9)
**Birth country (%, *n*)**	
Australia	56.2 (9)
United Kingdom	31.3 (5)
New Zealand	12.5 (2)
**Aboriginal or Torres Strait Islander (%, *n*)**	
No	100.0 (16)
**Member check completed**	
Yes	6.2 (1)
No	93.8 (15)
**Role**	
Parent	62.5 (10)
Staff	37.5 (6)

### Data Collection

Interviews were conducted using a semi-structured discussion schedule that detailed questions designed to solicit insights to address the overarching research question (see Supplementary Appendix for full interview schedule). The interview script was drafted and reviewed by the researchers (KG, MW, SR, MS, and RS), who have proficiency in qualitative research and/or the research topics. Key theories pertaining to school-based masculinity development were also drawn upon to guide the framing of interview questions (such as theoretical understanding of the “perception gap” between boys’ adherence to masculine norms and their belief that others expect them to conform to these norms; alongside theoretical understanding of masculinities as socially conditioned and learned behaviors that are influenced by context).

Teachers’ and parents’ perception of the experience of masculinity was gaged *via* questions relating to three topics: being and becoming a young man (e.g., “*Some people think there are ‘unwritten rules’ about being a man. If you think this is the case, what unwritten rules do you think exist?”*; and the characteristics constituent of “positive masculinity” (e.g., “*What sort of characteristics do you think describes a ‘positive masculinity’?*”). Additionally, teachers’ and parents’ perception of school-based strategies to promote positive masculinities was assessed *via* questions probing the role of the school and potential areas of school-based promotion of healthy masculine identity development (e.g., “*How could the school help students navigate moving from boyhood to manhood in a positive way?*”).

### Procedure

The study received ethics approval from the University of Melbourne Human Research Ethics Committee (ID number: 1852421), with data collected in November, 2019. Two researchers conducted the interviews (KG and MW). Both interviewers were Research Assistants with BA (Hons) degrees, and prior experience with qualitative data collection and analysis. Participants were emailed the plain language statement and consent form to read and complete before the interview. The interviewer re-established participants’ understanding of the study and consent before commencing the interview. Participants’ anonymity and confidentiality of their responses were also reiterated to promote honesty and transparency during interviews. Individual interviews were conducted at the school, as a familiar setting for participants (*n* = 12), or over the phone where participants were unable to attend a session in-person (*n* = 4). Interviews ranged from 21 to 43 min in duration. The audio recordings were transcribed verbatim by an online transcription service and checked for accuracy by the researchers. After the interview was complete, researchers offered member checking (i.e., the process of re-engaging participants to review and edit their interview transcripts) which was taken-up by one participant with no changes made.

### Data Analysis

Thematic analysis (TA) was implemented to generate themes in the study data (Braun and Clarke, 2006). Inductive TA was employed by the coding researcher (KG), where analysis was largely driven by the content of the interview transcripts, though data were examined through the coding researcher’s disciplinary knowledge of gender and masculinity theories. The findings were advanced conceptually through applying Connell’s (1995) masculinities framework as a means to theorizing the findings and connecting with relevant existing research, particularly in terms of masculine hierarchies and hegemonic masculinities. With the research question in mind, the individual interview transcripts were read, highlighting excerpts and making jottings about preliminary interpretations and descriptive labels (186 labels initially) to organize the data. The parent interviews were analyzed using constant comparative methods to distil patterns and diversity within and across participant perspectives. The teacher interviews were similarly analyzed and the patterns compared with the overarching perspectives shared by parents. As the analyses continued codes were subsumed and the descriptive labels were developed to more fully pre-empt the thematic findings. The research team met to discuss their interpretations and consensus was driven both in terms of the weighing of the themes and the illustrative quotes that were used to anchor the findings in the interview data.

### Interviewer and Analyzer Characteristics

It is important for interviewers and qualitative data analysts discuss relevant personal information that might inform our approach to interviewing and analysis (Elliott et al., 1999). The lead interviewer and co-data analyst, author KG, is a Caucasian Australian female in her mid-20’s. She attended a high-fee co-education high school in Melbourne. To contextualize her schooling experience, she opted to attend a co-education school rather than a single-sex school given public discourse of the perceived environments in single-sex schools. Her research background includes boys’ and young men’s mental health, masculinity, and mental health in elite sports. Additionally, author MW (co-interviewer and data analyst) is a 27-year-old, Caucasian, gay Australian male. He attended a co-educational independent school in regional Victoria. He approached the interviews and data analysis process with clear awareness of current discourse regarding the potential for single-sex boys’ schools to perpetuate traditional embodiments of masculinity, whilst also recognizing the need to promote diversity and difference among school-based masculinities. His research background includes boys’ and young men’s embodiments of masculinity, and intersections with mental health and help-seeking experiences.

## Results

Five overarching themes were generated from analysis of the interview transcripts. These themes are presented in Table 2, categorized as influencing factors and strategies for development.

**TABLE 2:** Summary of themes with exemplar quotes.

Category	Theme	Exemplar quote
Influencing factors	Public and private masculinities	*I feel there’s a lot of mixed messages that they’re getting*… *And what he’s picking up on, I’m never quite sure. I mean, his behavior that I see is fine, but I don’t know how he is then when he’s with his peers* [Mother, 003]
	Prevailing Australian masculinities	*Somebody that’s admired for his physicalities, his sporting abilities. And often that’s where we start with masculinity* [Father, 002]
	Private boys school culture	*I think given the environment, the historical environment of [the school] is not dissimilar to other all boys environments in terms of the culture of the place. I think, like I said, we’re at the beginning of a shift* [Male teacher, 016]
Strategies for development	Exposure to positive masculinities	*Having role models and maybe being more clear on these are the behaviors [we want to avoid], and probably be more explicit in that. Sometimes kids don’t pick it up, unless you’re actually explicit with that language or that behavior.* [Male teacher, 008]
	Transforming masculinities	*He [son] felt it would be really good if [programming] was consistent throughout the time that they were there* [Mother, 003]

*Influencing factors* encompasses three themes related to teachers’ and parents’ perceptions of the state of masculinities for boys and young men. Within this category, themes drew on participants’ perspectives about the key gendered influences on male students. Participants commonly offered concessions that some potentially negative influences could not be controlled. Rather the focus was on recognizing the potential for an array of exposures for shaping masculinities in boys. Three themes, *public and private masculinities*, *prevailing Australian masculinities* and *private boys’ school culture* were inductively derived to distil the factors influencing masculinity development in greater depth. The final two themes, categorized under *strategies for development*, summarize teachers’ and parents’ perspectives for prioritizing healthy identity development within the school, which can be in conflict with conventional academic priorities. This was coupled with discussion of practical initiatives to establish a positive developmental trajectory for male students. Across these themes, there was consistent support among parents and teachers for greater educational programming around development of positive masculinities.

### Influencing Factors

#### Public and Private Masculinities

There was a perception amongst participants that the impact of masculine norms can shift depending on social settings and group dynamics. Often, participants would report that when students were alone, they were typically more authentic than when they were in a group setting. It appeared that the nature of social messaging, including where and how boys learn masculine norms, or perceive pressures to act in certain ways relative to the particular context of interaction, could influence expressions of masculinity. The idea that boys and young men need to “*wear a mask*” in order to protect their masculine standing was commonly mentioned; an idea that has clear implications for the exposure of vulnerability needed to facilitate shifting traditional masculine norms in the school context.

*I think that you kind of do have to wear a bit of a mask at school sometimes to just fit the norms* [Mother, 005]

*I feel there’s a lot of mixed messages that they’re getting*… *And what he’s picking up on, I’m never quite sure. I mean, his behavior that I see is fine, but I don’t know how he is then when he’s with his peers* [Mother, 003]

Participants discussed how in a group of males, the loudest voice often dominates to message and manage masculine norms, particularly regarding upholding the hegemonic “tough guy” archetype referenced by Connell and Messerschmidt (2005). Many participants indicated that males who do not conform to, or are complicit in upholding this standard can experience consequences for their wellbeing. Likewise, participants recognized the impact that different social settings can have on boys’ and young men’s behaviors, for instance in the school relative to the home. As one teacher noted, the school environment is “*a little bit different to society*” [Male teacher, 010].

*It’s a bit of a self-fulfilling prophecy really. That some of them feel that to be accepted and to be the most sort of manly of men, they need to be on the footy team and they need to do*… *all those sort of traditional male stereotypes* [Female teacher, 006]

*There’s still the hidden expectations of boys with each other, and from their parents having the expectation of their boy which then feeds into the school* [Male teacher, 010]

Participants also spoke about the role of the so-called “group mentality” in influencing displays of masculinity by male students, where participants often described first-hand experiences observing students’ adherence to traditional masculine norms in group settings. Moreover, participants recognized the flexibility among male students to align with more traditional masculinity norms depending on social context, with a key determinant appearing to be the group nature of the school environment. This may represent a desire among teachers and parents for knowledge of how to encourage male students to feel comfortable enacting positive masculinities, such as disclosing distress or a need for support, irrespective of context.

*I think when they get into a group, it’s very different. Yeah. That changes the dynamics. They obviously play off each other and they change their behaviors. Whereas, sometimes I’ll see a different boy versus a boy in a group* [Male teacher, 008]

*I think when they get into bigger groups they can fall into those sort of more stereotypical old-school male behaviors*… *And I know all those boys are wonderful and amazing and much more authentic in smaller groups, than large groups* [Female teacher, 006]

#### Prevailing Australian Masculinities

Participants often discussed how Australian cultural norms are incited in the school context, often with reference to traditional, and/or hegemonic masculine norms (Connell and Messerschmidt, 2005). Described were the ways in which sporting culture, the “blokey” archetype of masculinity and male teasing humor can impact students’ experiences. Irrespective of whether male students were complicit in sustaining this masculine culture, participants recognized the “sporty young man” as the prevailing norm.

*Look, it’s a boys’ environment, so they’re very boysy* [Father, 007]

Participants also recognized how the sporting culture in Australia norms athletic achievement, prizes physical prowess, and celebrates success or dominance over one’s competitors as idealized hegemonic masculine qualities; a process which according to one father represents an “*unwritten rule around physicality, in a sporting sense*” [Father, 014]. Sports that are perceived as more physically challenging or aggressive (e.g., Australian football or rugby) are favored over sports or other activities that are deemed less so (e.g., badminton, the arts).

*You have to kind of engage in the banter. Which would be in Australia primarily banter about sport, so if you’re not interested in that, you can’t fully participate* [Mother, 011]

Even when one does not participate in physically demanding sports, there is an expectation that male students enjoy the spectacle, and express their opinions and authority in discussions about such masculine sports. This echoes Connell and Messerschmidt’s (2005) discussion of complicit masculinities as embodied by individuals who sustain such hegemonic norms, even if (and perhaps especially when) they do not fit this archetype. In schools, the sporting “blokey” student is revered; with the superiority of their status regularly appearing to permeate and predominate within the entire school’s masculine milieu.

*The first thing I learned when I came here was that a lot of the prefects were quite sporty. And then if the prefects that weren’t as sporty were seen as quite dorky* [Female teacher, 004]

A small number of participants indicated how culturally sanctioned male humor can be instrumental in the construction of “masculine” behaviors. Mentalities necessitating that boys “take a joke” or “join the banter” could be used to excuse poor conduct, or pressure an individual to follow the group in relation to masculine norms. These ideas expose the perceived risk of peer-level consequences for boys and young men who might communicate their individual conflict with these norms.

*Because it’s that whole pervasive, “Oh, you can’t take a joke,” mentality. [In] Australian men that is a big problem.* [Mother, 005]

Normative humor and jovial behavior among Australian males were recognized by participants as imparting a diversity of influences on males and those around them, ranging from inciting fun and healthy competition to imparting negative consequences for those young men who were perceived as oversensitive and fail to “fit in” with the dominant male archetype within the school. It was implied by some participants that this prioritization of banter could cloud boys’ and young men’s perception of any warning signs pertaining to mental ill-health in their peers.

*It can be a great part of being a friend and being a boy and being a man. That playfulness and that banter, but it can also become damaging as well* [Male teacher, 016]

*There would be boys there who probably weren’t even thinking about what they were doing. They were just joining because it was funny* [Mother, 005]

#### Private Boys’ School Culture

Most teachers and several mothers discussed the presence of a narrative regarding the nature of private boys’ schools which hinted at the potential risks of this context in the construction of adolescent masculinities.

*If you listen to the social narrative, there’s not a lot of optimism about boys’ schools at the moment* [Female teacher, 004]

Whilst participants did not indicate any direct concerns, several interviewees acknowledged questions among the Australian community as to the role of private boys’ schools in the potential acculturation of problematic embodiments of masculinity, and the role that this culture can play in explaining reticence toward exposing vulnerabilities *via* help-seeking among boys and young men. Several participants indicated an awareness of how this view can be mirrored by broader society, though believed that it was not reflective of the school’s individual ethos or their direct experience.

Whilst enrolling their sons in the school from which participants were sampled was a choice, some parents discussed their initial hesitancy to send their sons to a private boys’ school, due to their awareness of concerns perceptions of prevailing masculinity norms that might be perpetuated by this environment. As one father [012] termed it, there was concern around his son getting “*sucked into*” a culture he might not necessarily feel individually comfortable with. From the mothers’ perspectives this concern was due to the potential negative impacts of a male-dominated space, whereas for fathers this was attributed to the perceived values of single-sex boys’ schools.

*A boys’ school wasn’t necessarily what I was going to choose for my kid, because I was worried about the toxic masculinity and the privilege.* [Mother, 005]

*When he first went to school, he went to a co-ed school. I didn’t want him to be like so many of the guys that I work with.* [Mother, 015]

*My big concern with this school was that it was very sporty*… *So I was concerned that [my son] might get sucked into that, but he isn’t.* [Father, 012]

Conversely, some teachers expressed that they felt social concerns around the impact of the private boys’ school environment may have been overstated, with one participant stating, “*I think they need to come here for a day and just actually see what happens here*” [Female teacher, 004]. Additionally, some teachers noted the benefits of a single-sex learning environment for boys, including the capacity for certain activities traditionally coded as feminine to become normalized when enacted by male students, highlighting the potential for single-sex schools to simultaneously challenge and reinforce traditional masculinity norms, depending on sub-context.

*I think it’s actually easier in one way to break down that mold at an all-boys school. It creates the space. There’s a lot of kids involved in drama, they actually get really passionate about it and arts and stuff.* [Male teacher, 010]

### Strategies for Development

#### Exposure to Positive Masculinities

Participants indicated that they valued the opportunity for male students to be exposed to varying embodiments of masculinity, in terms of role models and *via* varied learning experiences in order to provoke internal exploration and self-determined identity development. Reflecting a degree of awareness of some of the core tenets of gender justice (Lingard and Douglas, 1999; Keddie, 2006), some participants voiced the importance of normalizing certain stigmatized traits or behaviors among males, such as expressing emotions, seeking help, and disclosing distress. This was supported both within and beyond the school context.

*If you can set the container right and boys can share and they can realize, “Oh it’s okay to feel like that.” Or they feel like that too. That’s normalizing, expressing your feelings and stuff like that* [Mother, 005]

*It is changing [sensitivity as a sign of weakness] as there is a greater awareness and honesty. And that comes with, I think, seeing more and more examples of people that are prepared to discuss challenges more openly.* [Father, 014]

Exposure to masculine role models that could serve as a circuit breaker to provide exposure beyond the microcosm of the private boys’ school environment was seen as a key mechanism through which boys might experience and learn the normalization of certain behaviors and qualities traditionally coded as anti-masculine.

*It’s very difficult because his environment at the moment is his family and his peer group, et cetera. He meets other people when we give him other experiences, but that’s fundamentally his world at the moment.* [Father, 007]

*I think maybe a bit more genuine community impact. And that has an important role to play in boys understanding that there’s more outside of going to a privileged school* [Mother, 015]

Strategies that were desired within the school included leveraging role models, such as senior students, staff and community members, who can model healthy embodiments of masculinity, alongside the need for space in the curriculum for novel and reflective learning experiences. Additionally, notwithstanding the lack of consensus among participants regarding what constitutes positive or healthy masculinities, or exactly what a healthy male role model might look like, parents and teachers consistently reported an appetite for greater intervention and educational programming within the school to encourage development of healthy masculine identities.

*The more we could do for boys in school to have these conversations and offer an alternative viewpoint from the thinking that they’re probably used to thinking is only going to be a good thing* [Mother, 005]

*I think what the school needs to give is as many opportunities as they possibly can so that the boys can sort themselves out.* [Father, 007]

*I think you’ve just got to keep showing a diversity of different. like, the right behaviors in a sense. That’s, I suppose, going to come to an agreement of what the right behaviors are* [Male teacher, 008]

Furthermore, participants valued opportunities for boys to develop autonomy and independence. It was noted by one father that future efforts by schools should aim to provide encouragement toward healthy identity development, without forcing boys to take any given path, such that this can be a self-determined process.

*There’s a certain degree of freedom that’s allowing boys to now take their own path. I think that there’s a nice progression there from here’s what we’re expecting through to now we’re watching you, to not watching you, but we’re giving you the freedom to go and express that and make your own decisions.* [Father, 002]

#### Transforming Masculinities

Equally expressed in discussions regarding social contexts, participants discussed the shifting and varied messaging boys received in school. Notably, parents indicated a marked shift from early high school, where there had been more space to focus on masculinities and identity development, to late high school, where the focus shifted largely to prioritizing academic achievement. The lack of focus on positive identity development in the last 2 years of school was perceived to detract from the school’s ethos, ostensibly reducing the focus on holistic identity development in favor of academic success. It was important to parents that the focus on healthy masculinities and identity development remain constant across all year levels in the school, and that learning opportunities were relevant to each year level’s needs and current gendering experiences. Such perspectives imply the need for mental health promotion efforts that span early- to late-high school, such that healthy and positive embodiments of masculinity can be consistently and longitudinally reinforced.

*But then next year, I guess he’s just going to spend the whole year fixating on exams.* [Father, 012]

*He [son] felt it would be really good if [programming] was consistent throughout the whole time that they were there* [Mother, 003]

Encouragingly, teachers commonly noted how the school’s culture had shifted toward holistic development and healthy masculinities in recent years, alongside the importance of consistently expressing that ethos to students and the community.

*The school has transformed and the messages it gives to the boys consistently and it’s deep rooted in our whole ethos and this school is very different and very powerful.* [Female teacher, 009]

Participating teachers also appeared to take pride in the fact that they were offering an environment to boys and young men wherein their success is defined not purely in terms of academics, but also in the extent to which students develop qualities characteristic of a “good man.”

*It’s changing a bit because schools put some outward facing stuff to say that, actually if you send your son here, we’re going to be focusing, we will have some focus on their wellbeing, on mindfulness at times, on a proper healthy identity* [Male teacher, 010]

*I think we’ve even taken the word successful out of a lot of our vocabulary at school. So we talk about being a good man rather than being a successful man* [Female teacher, 009]

## Discussion

The aim of this study was to explore parents’ and teachers’ perceptions of masculinity development in an a single-sex independent school context, and their views regarding priorities for school-based developmental programming designed to encourage healthy expressions of masculinity, to inform mental health promotion interventions. The first three themes, categorized as *influencing factors*, detail teachers’ and parents’ views on the nature of masculine identity development and performance in the school context. Commonly noted was the difference between so-termed public and private masculinities (Hearn, 1999), where often the single-sex school context can serve to facilitate the group-level adherence to traditional masculine norms. Additionally, the latter two themes were categorized as *strategies for development*. Teachers and parents noted the importance of boys’ exposure to positive masculine role models. Finally, the prioritization of activities and programming supportive of positive masculine identity development was perceived by parents to be in conflict with the focus on academic success during latter years of schooling.

### Influencing Factors

Results highlighted that participants were acutely aware of many of the mechanisms underpinning the reinforcement of specific traditional masculinity norms, discussed by Connell and Messerschmidt (2005) as indicative of hegemonic masculinity, such as sporting prowess and emotional restrictiveness within the school context. Teachers and parents frequently commented on the disconnect between public and private masculinities, and in order for boys to fit in at school, they must adopt a “mask” that is complicit in sustaining normative behaviors within the all-boys school context. These norms included sporting prowess and “blokey” teasing humor; discussed in prior work exploring “laddish” cultures among boys at school and the extent to which these codes of behavior problematize engagement with schoolwork (Jackson, 2010). Notably, most participants also recognized that masculine norms were most likely to intensify, or exaggerate, in group situations. A theoretical explanation is offered in precarious manhood theory, which posits that manhood is a status that must be continuously and visibly demonstrated and defended (Vandello et al., 2008). Boys may exaggerate socially gender-typical behaviors when their actions are visible to (and likely judged by) their peers. This finding also accords restrictive masculinities detailed by Reigeluth and Addis (2016), in terms of a policing of masculinity, which serves as instrumental in the gender role socialization of boys and young men at school.

Notwithstanding participants’ perception of the dominance of certain masculine norms within all-boys’ schools, inherent in their discussion of public and private masculinities is the understanding that boys and young men *can* be flexible in their adherence to masculine norms. One example of this is the perception of normalization of behaviors traditionally coded as “feminine” within the all-boys school context, such as drama and engagement with the arts. Keddie (2006) discusses the imperative of allowing boys opportunities to broaden their enactment of masculinities in school contexts, and facilitation of engagement with traditionally non-masculine pursuits is an important vehicle in this process. Notwithstanding this, perhaps all-boys school environments represent a particularly salient force that polices adherence to traditional norms; yet in spite of this, parents and teachers understand that often behaviors reflecting these norms can represent a state-based means to prove one’s masculinity to peers, rather than being indicative of fixed behavioral traits. These perceptions of group-level upholding of masculine codes of behavior were also observed by teachers interviewed in Jackson (2010, p. 507), where teachers reported boys’ and young men’s adoption of a “pack mentality.” Echoing established scholarship regarding how gender justice can be advanced in schools (Keddie, 2006), such findings speak to the value of group-based interventions for boys and young men, where space can be provided to expose the power and policing of peer expectations for their embodiments of masculinity (King et al., 2021; Rice et al., 2021).

This idea has been implemented in previous gender-transformative programs focused on help-seeking intentions for suicidality (Calear et al., 2021), and reducing support for the use of violence in relationships (Banyard et al., 2019). Past research has found evidence of a disconnect between that which boys and young men believe individually and what they anticipate their friends believe concerning expected masculine behaviors (Irvine et al., 2018). Building on this, recent theoretical work has also operationalized positive masculinity, with authenticity as a key component (Wilson et al., 2021a). The tension between public and private masculinities has also been reported in prior research with high school and tertiary student young men (Reichert, 2001; Edwards and Jones, 2009). This can be viewed as a conscious self-fashioning, where boys may purposely adopt a public persona that aligns with the acceptable performances of masculinity in the school (Kehler and Martino, 2007). Prompting male students to interrogate how and why their behaviors or attitudes oscillate between contexts may promote a shift to more consistently authentic behaviors, unimpeded by restrictive and fixed gender norms. As such, facilitating an environment for boys to critically reflect on their behavior and understand the extent to which the norming of certain behaviors can be helpful or harmful, is important to helping boys establish a self-determined, prosocial masculine identity. There are current efforts underway to evaluate programs seeking to achieve this end (King et al., 2021).

Whilst these findings reinforce the value of group-based mental health promotion interventions grounded in shifting masculine norm adherence, the acute awareness among parents and teachers of the mechanisms underpinning reinforcement of traditional masculinities, signals opportunity to more directly involve parents and teachers in reinforcing the group-based learning imparted *via* such programs. Indeed, some comments from fathers in particular appeared to standardize the existence of masculine norms within the school context (e.g., “*look, it’s a boys’ environment, so they’re very boysy*”). This potentially signals a sense of perceived intractability of school-based masculinity norms among some parents (particularly fathers). However, fathers also expressed concern about their sons getting “*sucked in*” to a culture of masculinity with which they did not necessarily agree. These results have implications for school-based initiatives aiming to shift masculinity norms, as it is clear that there is scope to upskill parents, particularly fathers, in their capacity to model healthy masculinity norms at home to reinforce any messaging imparted *via* school-based intervention. Past research aiming to improve youth mental health has involved parents *via* improving their mental health literacy in attempt to model positive attitudes toward help-seeking and the norming of mental health challenges, particularly in adolescence (Hurley et al., 2018). Yet given the instrumental role of fathers in modeling masculinities (Fellers and Schrodt, 2021), there is potentially scope to develop complementary home-based initiatives for families, such that any gains in shifting masculinity norms in relation to mental health and promotion of help-seeking at school, are carried through at home.

Conversely, given that sporting prowess is prized masculine capital within school contexts, there is also scope to more directly involve a range of teachers in programming to promote a diversity of perceived masculinities among students. This is important in light of the role physical education teachers can play in inadvertently perpetuating masculine norms, particularly regarding male teachers (White and Hobson, 2017). Given exposure to positive role modeling was a desired development strategy among participants, future research may seek to uncover ways in which parents and teachers can congruently occupy role model positions in their everyday actions with sons/students. This call is also reflected in the 2021 Insights Report from the *Man Cave* program: a school-based workshop-style intervention for boys and young men focused on deconstructing masculine norms as a means to promote mental health (Defina et al., 2021). Specifically, facilitators communicated that it is “critical [we parents] look at our own actions and words and ensure they align with the messages we’re trying to pass onto [boys and young men].” This might be an important avenue to bolstering gains made *via* student-level interventions promoting healthy masculinities in the context of an all-boys school, where peer-level policing of masculinity expression is thought to be most salient (Kirk, 2000; Kehler and Martino, 2007); particularly in all-boys schools (Kirk, 2000).

### Strategies for Development

School-based health promotion programs targeted for boys and young men increasingly incorporate a focus on reducing adherence to traditional masculine norms, by offering group-based activities designed to encourage reflection on links between masculinity norms and mental health and behavior (Banyard et al., 2019; Claussen, 2019). Yet foremost among our participants was a desire for boys and young men to have greater exposure to positive masculine role models. The perception was that this could short-circuit negative psychosocial development and help boys to appraise a positive masculine blueprint to strive toward. Whilst gender-transformative developmental programming is increasing in implementation (Gwyther et al., 2019), and role modeling of help-seeking masculinities was incorporated in the *Silence is Deadly* program (Calear et al., 2017), there is a sparsity of academic research exploring the direct effects of positive role modeling on masculine identity development among boys and young men. One qualitative study addressing emotional mentoring relationships between male youth and adult men highlighted potential for facilitating flexibility in the enactment of masculinity norms such as self-reliance and emotional restriction (Spencer, 2007). Research has also highlighted that children’s perceptions of fathers’ embodiment of traditional masculinity is linked to reduced father-child relationship closeness and satisfaction (Fellers and Schrodt, 2021). There is a dearth of research that explicitly articulates masculine role models that might facilitate boys’ and young men’s adoption of positive masculinities, and by extension, promote mental health behaviors. For example, future research could explore whether a positive masculine role model should embody the inverse of so-termed “toxic” masculinities (Ging, 2019), or whether *both* the embodiment of positive masculinity (Wilson et al., 2021a) and critical reflection on the harmful nature of traditional masculinities is required to facilitate boys’ and young men’s mental health. Further research is also needed to understand how to effectively implement positive role modeling for boys and young men in order to achieve lasting change, given a specific focus on father-son dyads appears scant in the available literature.

Furthermore, results demonstrated a slight disconnect in the extent to which teachers and parents see healthy masculine identity development prioritized in tandem with academic success at school. Some developmental programs for boys and young men have involved parents (Wilson et al., 2021b), yet further research is needed to clarify parents’ expectations around how best to support both positive identity development ventures alongside academic outcomes. This is especially important given past evidence of the perception among teachers that boys and young men prize and prioritize their masculine social standing through the rejection of academic priorities (Jackson, 2010). There is therefore the risk that, without involvement of parents and teachers in cohesive efforts to shift adherence to masculinities, both academic engagement and healthy masculine identity development will be trumped by boys jockeying for peer-group social standings within traditional masculine hierarchies and collectively protesting subordinate student status.

## Limitations

To our knowledge, this is the first study to provide an in-depth examination of the perspectives of parents and teachers on school-based masculinity development and avenues for improvement in school-based developmental programming. Several study limitations nevertheless require acknowledgment. Firstly, the focus on one school and a relatively small participant sample limits the generalizability of the results, especially considering results pertaining to parents who had the means to choose an independent high-fee school for their sons. The concept of a school-based intervention designed to promote help-seeking *via* shifting masculinity norms is itself an initiative that has thus far primarily been studied among schools with the resources to implement such programs alongside established programming. As such, the extent to which these results generalize to families associated with less-resourced schools is limited and democratization of this work is needed. Additionally, the nature of the sampling may have limited perspectives to those who were interested and invested in the study area; those that did not respond to the email invitation may hold differing opinions than those identified in this sample. Similarly, participant characteristics may be reflected in the results, namely, the focus on Australian culture could have emerged due to the large percent of participants who were born in countries other than Australia, where differences between countries may be salient. Additionally, as all participants identified as heterosexual, the present findings should be considered in the context of a heteronormative view of masculinity, with potentially limited applicability to sexual minority parents and teachers. We also acknowledge that we approached the analysis from a predominately *cis*-heteronormative view of masculinity. Whilst this helps to understand the results in terms of existing scholarship in the field that also adopts this frame, there is an inherent risk that continuing to apply this lens limits the necessary exploration of diversity and difference in masculinities that is needed to advance gender justice efforts more broadly, and norm embodiments of masculinity beyond the traditional frame. A limitation also concerns the transparency of participants’ responses. For parents, they might have pre-empted judgment from the interviewer or the research team, particularly concerning their discussion of decisions regarding sending their child to an all-boys school in light of societal discussion regarding the role of this environment in the potential acculturation of harmful embodiments of masculinity. Whilst there was no direct evidence of any censorship occurring during interviews, it is nevertheless a possibility and should be considered when appraising these results. A similar caveat concerns participating teachers.

## Future Directions

Future research directions arising from our results have been summarized in Table 3 below.

**TABLE 3:** Recommendations for future research.

Category	Recommendations
General recommendations	More diverse participant sampling to allow explorations between masculinities, mental health promotion and other social subgroup memberships (i.e., race, class, gender, sexuality).
	More direct exploration of links between masculinity norms, help-seeking and mental health symptom profiles (e.g., whether masculinity carries differential influences on help-seeking for anxiety relative to suicidality), and how programming to promote positive masculinities can apply across symptom profiles.
Influencing factors	Qualitative research with parents and teachers to better understand their (inadvertent) role in perpetuation of traditional masculinity norms per-context, particularly in relation to mental health and help-seeking.
	Qualitative group-based research with boys and young men to better identify the specific contexts and social mechanisms (e.g., the “perception gap”) that lead to perpetuation of traditional masculine norms, even when this is not conducive to wellbeing.
	Co-design curriculum for parents and teachers that complements programming for boys and young men, focused on modeling healthy embodiments of masculinity, alongside promoting reflection on inadvertent reinforcement of traditional masculine norms.
Strategies for development	Further research to define the characteristics of “healthy masculine role models” and explore the kinds of exposure needed (e.g., story-telling, experiential programs) to achieve positive benefits to promotion of positive masculinities among boys and young men.
	Work with school leadership to explore implementation avenues for promotion of school-based positive masculinities alongside, but not at the expense of, academic pursuits at school.

Foremost, future studies should aim to sample participants from varied settings, including rural, co-educational, and moderate to low socio-economic status schools. Such research will allow exploration of the intersections between masculinity and race, sexuality, economic status, and culture. Accurate and diverse representation of the current masculinized experiences of boys and young men will ensure future initiatives are aligned with the needs of particular sub-populations. This is particularly important concerning the experiences of boys identifying as transgender or non-binary masculine, where the gendered structure of an all-boys school is likely to give rise to particular effects on identity formation and mental health. Additionally, future research implementing programs such as *Silence is Deadly* and other initiatives focus on shifting embodiment of masculinities, would do well to more directly explore the extent to which these programs are shifting masculinity norms, and how peripheral parties such as parents and teachers can be involved in these programs to facilitate consistent messaging across contexts. Additionally, whilst existing programs have broadly focused on reducing adherence to traditional masculine norms as a conduit to promoting help-seeking in the context of suicidality, more direct exploration of links between masculine norms, likelihood of help-seeking, and different mental illness symptom profiles is needed. For example, the extent to which self-reliance interacts with anxiety relative to depression in reducing likelihood of help-seeking remains largely unexplored.

## Conclusion

The varying impacts that schools can have as an agent in, or site for, the production of masculinities were highlighted in the perceptions of the teachers and parents. The impact of social contexts, cultural norms, and community narratives were prominent themes in the interviews. Understanding these factors, participants identified key strategies to promote healthy masculine identity development within and beyond the school context. Notwithstanding this, there is clear scope for greater involvement of parents and teachers in the aspects of health promotion interventions that rely on and reflect healthy expressions of masculinity. If we are to assume health promoting outcomes for boys and young men are mediated by embodiment of prosocial masculine norms, then accounting for and involving *all* agents of influence in this process would be an advantageous step toward bolstering the potential wide-ranging benefits.

## Data Availability Statement

The datasets presented in this article are not readily available because provision of original data was not stipulated in the ethics approval for this study. Requests to access the datasets should be directed to SR, simon.rice@orygen.org.au.

## Ethics Statement

The studies involving human participants were reviewed and approved by The University of Melbourne Human Research Ethics Committee. The patients/participants provided their written informed consent to participate in this study.

## Author Contributions

MW led manuscript drafting and contributed to data collection and analysis with KG. MS supported data analysis and the manuscript revision. RS led funding acquisition, and supported study conceptualization and the manuscript revision. JO guided data analysis and manuscript revision. KC supported data collection and contributed to the manuscript revision. SR supervised all aspects of the study, supporting data analysis, and the manuscript revision. All authors contributed to the article and approved the submitted version.

## Conflict of Interest

The authors declare that the research was conducted in the absence of any commercial or financial relationships that could be construed as a potential conflict of interest.

## Publisher’s Note

All claims expressed in this article are solely those of the authors and do not necessarily represent those of their affiliated organizations, or those of the publisher, the editors and the reviewers. Any product that may be evaluated in this article, or claim that may be made by its manufacturer, is not guaranteed or endorsed by the publisher.
