# Supplementary material for: Exploring Teacher and Parent Perspectives on School-Based Masculinities in Relation to Mental Health Promotion
Source: Front Psychol. 2022 Jun 13;13:864124. doi: 10.3389/fpsyg.2022.864124 (PMC9235819; doi:10.3389/fpsyg.2022.864124)
Supplement: Supplementary file 1 [file Data_Sheet_1.DOCX]

**Appendix A**

**Semi-structured interview schedule**

“Thank you for giving your time today to take part in this study. What will happen is that I will ask you some questions about the *Making of Men* camp that was held last November (2018), and some broader questions about what it is to be a young man in today’s world. Before we start, do you have any questions?” [Double check informed consent has been attained].

If you’re ready I’ll start the audio recording.

**Area 1 – Being a ‘man’ and Masculinity**

1. Some people think there are ‘unwritten rules’ about being a young man. If you think this is the case, what unwritten rules do you think exist?
   1. What are your opinions about these rules?
2. In your opinion what does it mean to be a ‘good’ man?
3. What, or who has shaped your ideas of what a ‘man’ is?
4. When do you think a boy becomes a man?
   1. What is the process involved?
   2. How does someone ‘know’ they are a man?
5. (For parents) - What experiences in your life have made you think about the kind of man you want your son to be?
   1. Tell me more about those experiences – what was going on, who was involved, how were you feeling?
6. What comes to mind when I say “positive masculinity”?
7. What sort of characteristics do you think describe “positive masculinity”?

**Area 2 – School-based initiatives**

So now that we’ve talked about your ideas about manhood, let’s chat about masculinity at the school, and what the school could be doing to encourage student’s development of positive manhood.

1. What pressures (if any) do you think the students might feel at school to act or show ‘manliness’?
   1. If you could see masculinity – where would you find it in the school?
   2. How is masculinity encouraged or discouraged by other students, teachers, staff?
2. How might these pressures change over time from primary to high school?
3. How could the school help students navigate moving from boyhood to manhood?
   1. How could teachers, or curriculum have a role to play here?
4. What would you like the school to do, or provide, to encourage positive masculinity?
   - 1. Prompts: Interactive activities, social events, experiential learning, education in curriculum, activities with other schools
5. What concerns, if any, have you had with the school supporting student’s development into young men?
6. Any final thoughts or comments?
